# The Case Book

**Published:** 1872-03

**Authors:** N. M. Burkholder

**Affiliations:** Harrisonburg, Va.


### ARTICLE III.

### *The Case Book.*

BY N. M. BURKHOLDER, D. D. S., HARRISONBURG, VA.

What is the greatest present lack of our profession, if it be not a more accurate observation, and a more careful examination? We have colleges, and journals, which are fully equal to the demands of the science. What we want in this relation, is a contribution to the one of good minds, and to the other of good matter—facts, not mistaken observations.

We have State Dental Societies, and National Dental Associations, most necessary aids to progress, I declare; but still only aids in proportion as they bring together men of accurate observation, and of accumulated observation. Otherwise these meetings must amount to little more than that which results in a vocal contest between neighboring chanticleers—"a dissipation of strength in a trial of wind."

Discernment of things as they are—not as they appear to be—is evidently the first step and only method of advance in the attainment of knowledge. But it is not the only step. In the domain of facts there are many so palpable as to present themselves to the eye of the most careless observer. Others are perceived only by the closest scrutiny. And there are still others which are reached by way of induction, from the observation and comparison of things already ascertained to be facts, and by the most exhaustive proof. How great and important is the proportion of these last in the volume of Medical Science. How have we discovered valuable methods of treatment of disease? Certainly not by the most careful observation of the conditions in any one case, following the administration of a drug or the performance of an operation; but by a judicious comparison of these closely observed after-conditions in a great number of cases.

Here one may rely upon the memory, if he would be careless, or if he would profit little by his experience. But so complicated will be the series of observations in any one class of cases, that he must record them, if he would bring out a complete view of each case in order to a close comparison. Otherwise many important facts are obscured or lost; the mental vision of the whole is contracted, and the conclusions are inaccurate.

I verily believe there is no step which promises so much for the advancement of Dental Science to-day, as a general adoption of some systematic method of preserving a compact view of all operations in practice, together with all observations necessary to a fair view of each case.

What is the secret of the importance attached to hospital reports, if it be not the system of accurate observation behind them, and the facility thus afforded for close and extensive comparison—such as would require many years for attainment, if indeed it could be reached at all, by the usual resort to scattering reports from *Dick*, *Tom* and *Harry?* And why may not the office of every dentist be a miniature hospital in this respect? What employment of time will ensure returns so great as that devoted to the preservation of our experience? By this method of accumulating observations, why may not one possess at middle age a stock of experience, otherwise not to be acquired in a long life?

I repeat that there would seem to be no proposition of more importance for the consideration of the dental profession to-day than that which asserts the duty of more carefully preserving our observations, in order to a clearer and more extensive comparison, and thus to the acquisition of *truth* on the questions with which we have to do in the interest of mankind. And I respectfully suggest it for their consideration, with the desire that all practitioners, especially the younger members of the profession, may be aroused to a just apprehension of its importance, and that as a result of its examination, that particular form of Case Book may be projected which shall be at once compact and comprehensive, and which shall most likely secure general adoption.

On the following page will be found a simple form of a Case Book which I have been using, may serve in some degree to illustrate:

| Date. | Name of Patient. | Age. | Residence. | Operation and Locality. | Superf'l Decay Removed, &c | Nerves Destroyed. | Fangs Filled. | FILLINGS. Gold. | Tin. | Amalgam. | Temporary. | Extracted. | Artificial Substitutes. | Fees. | Received. |
|---|---|---|---|---|---|---|---|---|---|---|---|---|---|---|---|
| 1872. | | | | R. app. L. sup'r Cent. inc | | | | 1 | | | | | | | |
| Feb 1 | A. B. | 20 | Harrison- | Post'r dep'r Gdg. surf. R. inf. 2d Mol. | | | | 1 | | | | | | 5.00 | 5.00 |
| Feb 1 | A. B. | 20 | burg, Va. | R. app. palato angle L. sup. lat. inc. | | | | 1 | | | | | | | |

| REMARKS. | RESULTS. |
|---|---|
| (This embraces nature of disease, description of decay, method of treatment or of operation, condition of the teeth and adjacent parts, character or structure of teeth, and of sal'y calculus—diathesis, and in general any facts which may have a bearing upon the case under treatment as to failure or success.) | (Left vacant, and condition or result of operation entered from time to time with dates affixed—say in five, ten and twenty years from date of treatment.) |

NOTE.—The second or lower heading is the continuation of the first on the right hand page of the open book. The pages should be large ledger size, in order to furnish spacious columns for "remarks" and "results." In filling these columns, with a view to facility of review, as well as of record, it is my rule to study to say the most in the fewest words. By the use of the familiar terms 'R." and "L." "app'l" "surf." for incisors. "Ant'r" and "post'r" "app'l" "surf," for the rest. "Grind'g" ("ant'r" and "post'r" depressions), buccal, lingual, labial, palatine surfaces and angles—in their various combinations greatly abbreviated, the locality of an operation can be fixed in an instant. This should be done with pencil and paper at the moment of its completion. At the close of each day entry should be made in the Case Book in full. In a large practice one may prefer to record observations in full only in one or two classes of cases, until a satisfactory number of cases has been collected, when another class of cases may receive his attention. The book may be kept in the order of the Merchant's Day Book, the names of patients following one another from the beginning of the book to the end, or each separate double page may be devoted to a family or to one patient, in either case using an index for reference to the names of patients.

